# Contribution of trunk swing to the performance of fixed-seat rowing

**DOI:** 10.3389/fspor.2025.1618375

**Published:** 2025-08-01

**Authors:** Renee Lafreniere, Matt Jensen, Tomislav Smoljanovic, James M. Wakeling, Marc Klimstra, Rebecca Thomas Orr, Courtney L. Pollock

**Affiliations:** ^1^ Department of Physical Therapy, University of British Columbia, Vancouver, BC, Canada; ^2^ Biomechanics & Performance Analysis, Canadian Sport Institute Pacific, Victoria, BC, Canada; ^3^ Department of Orthopaedics, University Hospital Center Zagreb, School of Medicine, University of Zagreb, Zagreb, Croatia; ^4^ Department of Biomedical Physiology and Kinesiology, Simon Fraser University, Burnaby, BC, Canada; ^5^ Exercise Science, Physical and Health Education, University of Victoria, Victoria, BC, Canada; ^6^ Para Rowing Commission, World Rowing, Lausanne, Switzerland

**Keywords:** para rowing, classification, trunk control, rowing ergometer, para sport

## Abstract

**Introduction:**

This study aimed to test the contribution of trunk swing to the performance during fixed-seat rowing in eligible and non-eligible (NE) para rowers. Assessment of trunk swing is used to classify para rowers with physical disability in Para Rowing (PR) 1 and PR2 rowers. PR1 rowers are classified based on the demonstration of impaired function of trunk swing.

**Methods:**

PR1, PR2, and NE rowers participated. Rowing ergometers were used in two different fixed-seating conditions, resulting in either (1) restricted trunk swing or (2) unrestricted trunk swing during the rowing stroke. Participants performed maximal effort 500 m pieces (race pace) in each seating configuration. Force production at the handle and fixed-seat rowing-specific trunk extension force was measured. Rowing performance measures were compared using a repeated-measures general linear model, including condition and group and an interaction between condition/group.

**Results:**

Only PR1 rowers generated greater trunk extension force during the restricted condition compared with the unrestricted trunk condition (*P* < 0.01). The restricted trunk swing condition resulted in a faster time to complete 500 m and minimal impact on force production for PR1 rowers. NE and PR2 rowers showed a significantly faster time to complete 500 m and greater stroke impulse (Ns) in the unrestricted compared with the restricted trunk swing condition (*P* < 0.01).

**Discussion:**

These results provide evidence-based reasoning for the classification of fixed-seat rowers. Contrary to PR2 and NE rowers, whose rowing performance was decreased due to trunk restriction, PR1 rowers' performance benefits from the trunk restriction.

## 1 Introduction

Classification of athletes in para sport aims to ensure that athletes with similar levels of sport-specific ability, in the context of permanent physical impairments, compete in a specific sport class, making the competitions as fair as possible (1–3). Classification of para rowing (PR) rowers is based on the mechanics of the rowing stroke. Force is generated in rowing (with the use of a sliding seat) using sequential movements that can be broken down into three parts: (1) the leg drive (push of the legs into extension), (2) trunk swing (extension of the trunk pivoting from the hip joint), and (3) arm pull (flexion of the elbows together with extension of the shoulders) (4). Of the three sport classes in PR, the PR3 sport class is for rowers who, regardless of their physical or visual impairment, can row using a sliding seat and, therefore, can produce force with the leg drive, trunk swing, and arm pull sport-specific movements of rowing (5). The PR2 sport class is for rowers who can perform and produce force using trunk swing (in addition to arm pull), but who are unable to use the sliding seat to propel the boat because of significantly impaired function of the lower limbs. The PR1 sport class rower, because of significantly impaired trunk function, produces force predominantly using the arms and shoulders during the arm pull of the stroke (5). Rowers without physical impairments or with an impairment that does not reach the minimal impairment for the sport are non-eligible (NE) for PR competition.

The PR1 rowers have various levels of impairment in performing the trunk swing; however, movement of the trunk would be primarily through the spine (e.g., flexion/extension through the lumbar spine) rather than rotation of the pelvis associated with hip flexion/extension. Previously, strapping to the backrest of the fixed seat was mandatory to stabilize the trunk of PR1 rowers at the mid-thoracic spine region (6). These regulations aimed to limit the amount of trunk movement during the rowing stroke, making the rowing stroke of all PR1 rowers similar looking. As the sport evolved toward including the abilities of a rower instead of restricting it, this strapping regulation was removed. PR1 rowers are now allowed to use seat straps as they wish to optimize their performance, aligning with the para movement to promote individualized equipment and seating configurations tailored to each athlete (7, 8). Accordingly, a greater range of incorporation of trunk movement is now evident while watching racing, and that causes questions about the robustness of the fixed-seat rower classification system (PR1 and PR2) and the fairness of the competition.

As the contribution of trunk swing to the rowing stroke underpins the classification of PR1 and PR2 rowers, this research aims to test the contribution of trunk swing to performance variables of fixed-seat ergometer rowing in PR1, PR2 rowers, and NE rowers. We hypothesize that PR2 and NE rowers will have significantly improved rowing ergometer performance during conditions of unrestricted compared with restricted trunk swing. Conversely, we hypothesize that in PR1 rowers, measures of rowing performance production will be significantly improved, but not the power production, under conditions of restricted trunk swing compared with unrestricted trunk swing.

## 2 Materials and methods

### 2.1 Subjects

Para rowers from all participating international federations, who competed as fixed-seat rowers in the World Rowing (WR) Gavirate International Para Rowing Regatta, Italy, in May 2018 or May 2019, were invited to participate in this study. All rowers were only able to participate once. NE rowers from a university rowing team served as a comparator group to inform the performance of fixed-seat rowing measures under experimental conditions in the absence of physical impairments (no reported impairment at any joint). Written informed consent was obtained from all participants, and participation was voluntary. This study was approved by the University of British Columbia Ethics Board (H19-01077). Participant characteristics including age, gender, mass, diagnosis, mobility, rowing experience, and training information were self-reported from each participant.

### 2.2 Experimental protocol

All PR participants were reclassified for the study specifically following the WR Classifier Manual, 2022 (available at the time of testing, prior to release) (5). All PR1 and PR2 rower data were collected prior to the start of racing at the regatta. Hip flexion and extension strength contribute significantly to trunk swing and were therefore measured as a study variable (collected during classification) using manual muscle testing (MMT) grading system ranging from 0 to 5 (1 refers to a flicker of muscle activity, whereas 5 denotes unimpaired muscle strength). All classifiers were certified WR Level 2 classifiers. Study classifiers were blinded to the participants' previous sport class. However, it is important to note that blinding was limited as it was not possible to control whether any of the PR1 or PR2 study participants were known to the classifiers in this study. Despite this study limitation, previous paperwork and sport class were not discussed until the end of the classification. A sport class of PR1 or PR2 was provided to each athlete. The results of this study classification were then compared for agreement with the athlete's current WR sport class.

**Figure 1:**
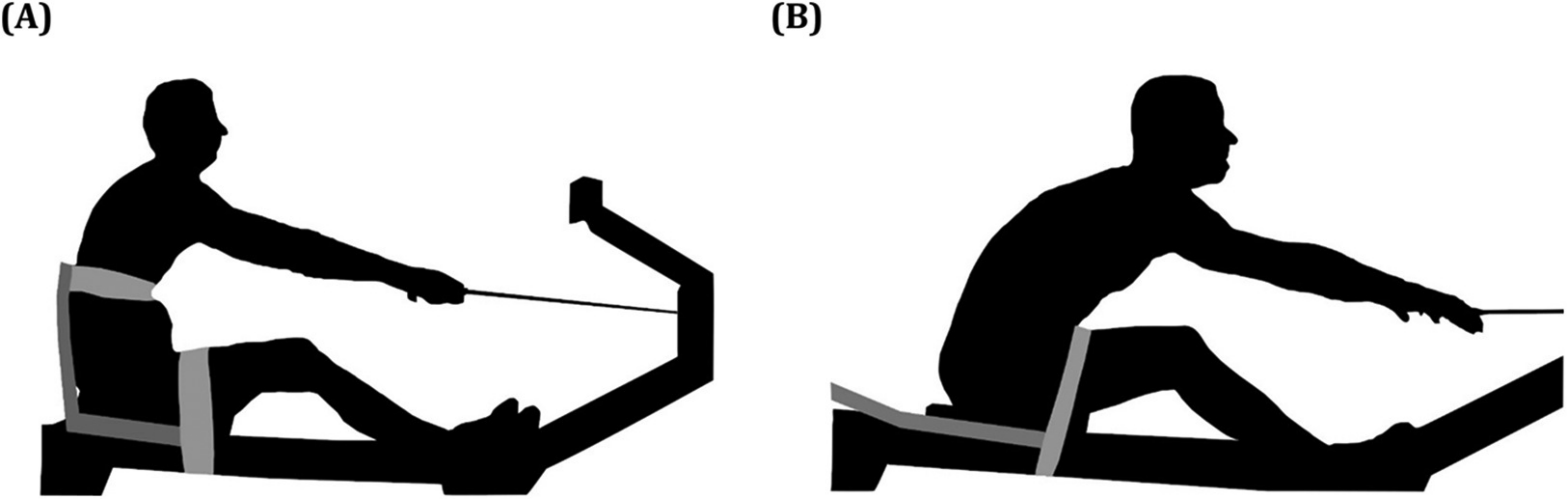
Experimental conditions testing the contribution of trunk swing to fixed-seat rowing on an indoor rowing ergometer. Two rowers at the catch—the initiation of the rowing stroke **(A)** during the restricted trunk swing condition and **(B)** the unrestricted trunk swing condition. The trunk strap, leg strap, and the fixed seat frame are outlined in gray. During the restricted trunk swing condition **(A)**, the seat back was positioned upright, and the trunk and leg strap fixation were in place for all rowers. During the unrestricted trunk swing condition **(B)**, the backrest was angled back to allow for each athlete to use their full available range of trunk swing as able. No trunk strap fixation was used.

Rowing ergometers used for rowing-specific dryland training [Model C Indoor Rowers with the performance monitor 4 (PM4), Concept2, Morrisville, VT, USA] were employed in two different conditions (Figure 1):

1. Restricted trunk swing position (Figure 1A)—rowers sat on a rowing ergometer with the addition of a fixed seat with a seat back (WinTech Fixed Seat, WinTech Racing, Shelton, CT, USA). Standardized strapping over the proximal thighs secured the athlete to the seat, and the trunk was secured to the seat back with standardized non-stretch strapping below the level of the chest of the rowers (mid-trunk) to restrict the trunk. This configuration restricted trunk swing pivoting about the hips. Seat back height was set for each rower so as not to block the extension of the arm pull during the drive. All rowers used the foot stretcher of the ergometer (amputees with prostheses as used for rowing).
2. Unrestricted trunk swing position (Figure 1B)—rowers sat on a rowing ergometer with a fixed seat. Standardized strapping over the proximal thighs secured the athlete to the seat; however, no strapping was used to secure the trunk to the backrest. This configuration allowed for unrestricted trunk swing if the athlete was able to. The backrest was angled back to allow for each athlete to use their full available range of trunk swing as able.

Rowers in each sport class were allocated codes specific to the order of testing. Allocation of order of testing code, within each sport class, alternated between “code A restricted first/unrestricted second” and “code B unrestricted first/restricted second,” to ensure half of the rowers in each sport class performed each order of conditions. Rowers unable to row in an unsupported sitting configuration due to limited balance and associated risk of fall were evaluated in the restricted trunk swing condition for all measurements, and their data were not included for further analysis.

Trunk function specific to trunk swing was measured in two different ways:

*Measurement 1 (dynamic): fixed-seat rowing performance measurement*. Participants performed a self-determined warm-up followed by two maximal effort 500 m pieces (race pace) on the rowing ergometer—one piece in each seating configuration. An Omega Miniature Load Cell (LC-202-1 K; capacity 4,448 N, Omega Engineering, Norwalk, CT, USA) was used to record the instantaneous force exerted by the participants on the chain of the ergometer (2,000 Hz). A range of forces of known magnitudes from calibrated weights was used to calibrate the load cell (linear correlation *r*^2^ = 0.99). The force signal was filtered using a second-order, zero-phase, Butterworth low-pass filter at 40 Hz. Timing/distance measurements were measured directly from the rowing ergometer. All PR1 and PR2 rowers were tested on the same ergometer for both experimental conditions, and in a different location, all NE rowers were tested on the same ergometer for both experimental conditions. The same Concept2 ergometer monitor and handle-mounted load cell were used across all experiments.

Performance variables (stroke rate, stroke length, impulse, peak force) were captured during the middle 50% of the 500 m piece, between 125 and 375 m, where performance is most consistent in experienced rowers (9–11), and where Concept2 ergometer power output recordings are more accurate (12). Stroke rate was measured as the cadence, or the number of strokes executed per minute (spm), and stroke length was measured as the length of the drive portion of the stroke (m), calculated internally by the Concept2 ergometer monitor. Impulse was measured as the average of the integral of force (Ns) produced over the drive phase recorded in all strokes by the handle-mounted sensor. Peak force was measured as the average maximum force (N) recorded in all strokes by the handle-mounted force sensor. The 500 m trial time was defined as the amount of time it took to perform each 500 m piece on the ergometer (s) measured internally by the Concept2 ergometer monitor.

*Measurement 2 (static): fixed-seat rowing-specific trunk strength measurement*. Isolated trunk extension strength, excluding force generated by arm pull on the handle during the stroke, was measured to test the impact of restriction of trunk swing, with fixation of the trunk to the seat back, on trunk extension strength production in a fixed-seat rowing position. Trunk extension force was quantified in Newtons (N) using hand-held dynamometry (HHD, microFET2, Hoggan Health Industries, UT, USA) in both seating configurations (restricted and unrestricted trunk swing positions) after completing each of the two 500 m ergometer pieces. HHD is not included in the classification of PR athletes. HHD was used to test the impact of the trunk strap to aid stabilization of the trunk as a lever during trunk extension. HHD has been shown to be a reliable and accurate way to measure isometric voluntary trunk strength (13, 14). These isometric measurements were taken at the mid-drive portion of the stroke, at which point peak force generation commonly occurs (15). The mid-drive position was measured approximately 90° of trunk extension (measured from the level horizontal axis, shoulders directly above the hips). With their arms across their chests, participants were instructed to build up gradually to their maximal trunk extension force. HHD was placed in the interscapular region of the back. Measurements were collected three times in each position, and the mean standard deviation (SD) is reported. For discussion purposes, we will refer to the trunk extension force variable as trunk extension strength to avoid confusion with the performance measurement of peak force at the rowing handle.

### 2.3 Statistical analyses

Descriptive statistics (including sum, mean, and SD) were used to describe participant characteristics. All statistical analyses were carried out using SPSS 19, and significance was set at an alpha of 0.05. Hip flexion and extension strength (right and left legs) parameters were compared using a multivariate general linear model (GLM) including group (PR1 and PR2). Rowing performance measures (500 m trial time, stroke rate, stroke length, impulse, peak force) and trunk extension strength were compared using repeated-measures GLM including condition (restricted and unrestricted trunk swing) and group (NE, PR1, and PR2) and an interaction between condition and group. Significant interactions were further explored using *post hoc* pairwise comparisons applying a Bonferroni correction (*P* = 0.02), and mean difference ± standard error (SE) and 95% confidence intervals (CI) are reported. All data are presented as mean ± SD, unless stated otherwise.

## 3 Results

### 3.1 Participants

Fifty-three rowers participated. Twenty-two PR1 rowers and 16 PR2 rowers consented to participate. Fifteen NE rowers without any physical impairments, recruited from a local university rowing club, consented to participate. One rower in the PR1 sport class was not able to complete testing in the unsupported sitting configuration due to safety concerns regarding loss of balance. Technical challenges related to the force sensor were experienced during data collection on the ergometer for an additional three rowers in the PR1 group. These four rowers' data were not included in the analysis. Participant characteristics are described in Table 1.

**Table 1:** Characteristics of the para rowiing (PR) 1 and PR2, and non-eligble (NE) rower groups.

Mean (standard deviation, SD)	PR1 (*N* = 18)	PR2 (*N* = 16)	NE (*N* = 15)
Age (years)	35.23 (11.41)	38.38 (11.67)	19 (0.76)
Gender (female/male)	6/12	6/10	7/8
Mass (kg)	63.89 (13.24)	67.50 (11.74)	74.35 (10.17)
Experience, mean (SD)			
Years rowing	3.01 (2.77)	6.69 (5.62)	2.43 (2.32)
Years comp	2.02 (2.05)	3.81 (3.08)	2.27 (2.28)
Training (hours/week), mean (SD)			
On water	7.96 (5.66)	10.31 (7.17)	12.25 (3.52)
Weights	2.59 (2.11)	2.72 (2.19)	2.46 (1.12)
Offseason Erg	4.73 (4.11)	3.59 (3.51)	3.7 (2.64)
Mobility (*n*, %)			
Wheelchair	17 (94%)	1 (6% full-time user) 3 (19% part-time user)	
Ambulatory with gait aid (i.e., crutches)	1 (6%)	10 (63%)	
Ambulatory unaided		5 (31%)	
Impairment/diagnosis (*n*, %)			
Complete spinal cord injury (SCI) total	11 (61%)		
High thoracic	2 (11%)		
Low thoracic	8 (44%)		
Lumbar	1 (6%)		
Incomplete SCI total	3 (17%)	2 (13%)	
Cervical	2 (11%)		
Low thoracic	1 (6%)	1 (6%)	
Lumbar		1 (6%)	
Lower limb amputation total	2 (11%)	7 (44%)	
Below knee		2 (13%)	
Above knee	2 (11%)	3 (19%)	
Bilateral lower limb		2 (13%)	
Hip arthrodesis	1 (6%)		
Multiple sclerosis	1 (6%)		
Poliomyelitis		2 (13%)	
Cerebral palsy		2 (13%)	
Peripheral nerve damage		1 (6%)	
Congenital limb deficiencies		2 (13%)	

No PR1 or PR2 rowers participating in this study had their sport class changed following classification for the study. Both hip flexion and extension strength, measured using MMT during classification, were significantly lower in the PR1 group compared with the PR2 group (Table 2, *P* < 0.001).

**Table 2:** Hip flexion and extension strength as measured with manual muscle testing (MMT) for the para rowing (PR) 1 and PR2 rower groups, mean ± SD.

Strength (MMT/5)	PR1	PR2
Right hip flexion	1 ± 1	3 ± 2*
Right hip extension	1 ± 1	4 ± 2*
Left hip flexion	1 ± 2	4 ± 1*
Left hip extension	1 ± 1	5 ± 1*

* *P* < 0.001 between group.

### 3.2 Trunk measurement 1: fixed-seating performance measurement

There was a significant interaction between condition and group for stroke rate, *F*_(2, 46)_ = 15.36, *P* < 0.001, partial *η*^2^ = 0.40. PR2 and NE rowers showed higher stroke rate during the restricted trunk swing condition than the unrestricted trunk swing condition (Table 3, *P* < 0.001). There was also a significant interaction between condition and group for the time to complete the 500 m piece, *F*_(2, 46)_ = 42.09, *P* < 0.001, partial *η*^2^ = 0.65. The time to complete the 500 m piece(s) was significantly faster in the unrestricted trunk swing condition compared with the restricted trunk swing condition for both NE and PR2 rowers (Table 3, *P* < 0.001). Conversely, the time to complete the 500 m piece was significantly slower in the unrestricted trunk swing condition compared with the restricted trunk swing condition for PR1 rowers. There were no between-group differences in the time to complete the 500 m piece in the restricted trunk swing condition (Table 3). However, in the unrestricted trunk swing condition, both NE and PR2 rowers demonstrated a significantly faster time to complete the 500 m piece than PR1 rowers (NE, mean difference ± SE −54.77 ± 9.79 s, *P* < 0.001, 95% CI −79.10 to −30.44; PR2, −47.41 ± 9.62 s, *P* < 0.001, 95% CI −71.32 to −23.50).

**Table 3:** Stroke rate (strokes/min) and 500 m trial time (s) for the para rowing (PR) 1, PR2 and non-eligible (NE) rower groups during unrestricted and restricted trunk swing conditions, mean ± SD, mean difference ± SE and 95% Confidence Interval (CI).

Group	Unrestricted trunk swing	Restricted trunk swing	Mean difference ± SE	95% CI
Stroke rate (strokes per minute)				
PR1	43 ± 8	40 ± 8	−2.48 ± 1.71	−5.92 to 0.96
PR2	33 ± 7	42 ± 8	8.95 ± 1.81*	5.30 to 12.60
NE	41 ± 6	51 ± 9	9.90 ± 1.87*	6.13 to 13.67
500 m trial time (seconds)				
PR1	180.05 ± 38.90	157.54 ± 26.49	22.51 ± 4.44*	13.57 to 31.45
PR2	132.64 ± 21.61	157.38 ± 37.45	−24.74 ± 4.71*	−34.22 to −15.26
NE	125.28 ± 15.46	157.46 ± 22.67	−32.18 ± 4.86*	−41.97 to −22.39

* *P* < 0.001, within group, between unrestricted and restricted trunk swing conditions.

**Figure 2:**
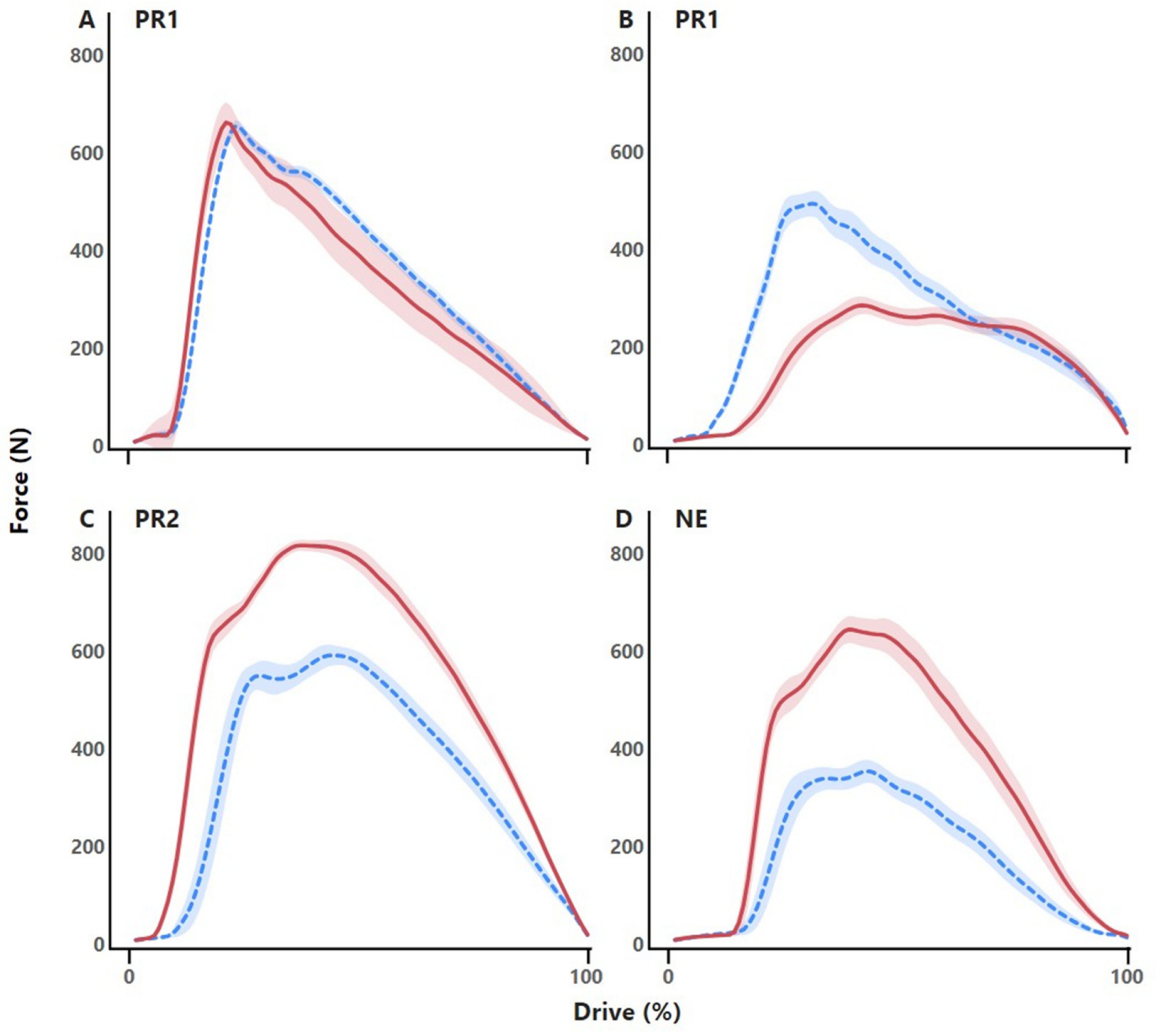
Representative force profiles of the drive (shown as % of drive time) from four different rowers; two rowers from the para rowing (PR) 1 (PR1) sport class **(A,B)**, one rower from the PR2 sport class **(C)** and one unimpaired rower from the non-eligible (NE) sport class **(D)** during restricted (blue, dashed mean line, shading indicating standard deviation) and unrestricted (red, solid mean line, shading indicating standard deviation) trunk swing conditions while rowing on the ergometer. PR1 rowers typically demonstrated either **(A)** similar force profiles or **(B)** a decrease in amplitude of force (N) during unrestricted trunk swing rowing compared with restricted trunk swing rowing conditions. PR2 rowers **(C)** and NE rowers **(D)** typically demonstrated increased amplitude of force (N) during unrestricted trunk swing rowing compared with restricted trunk swing rowing conditions.

PR2 and NE rowers typically demonstrated increased amplitude of handle force profile during unrestricted trunk swing rowing compared with restricted trunk swing rowing (Figure 2). Conversely, PR1 rowers typically demonstrated either similar or decreased force profiles during restricted trunk swing rowing.

**Figure 3:**
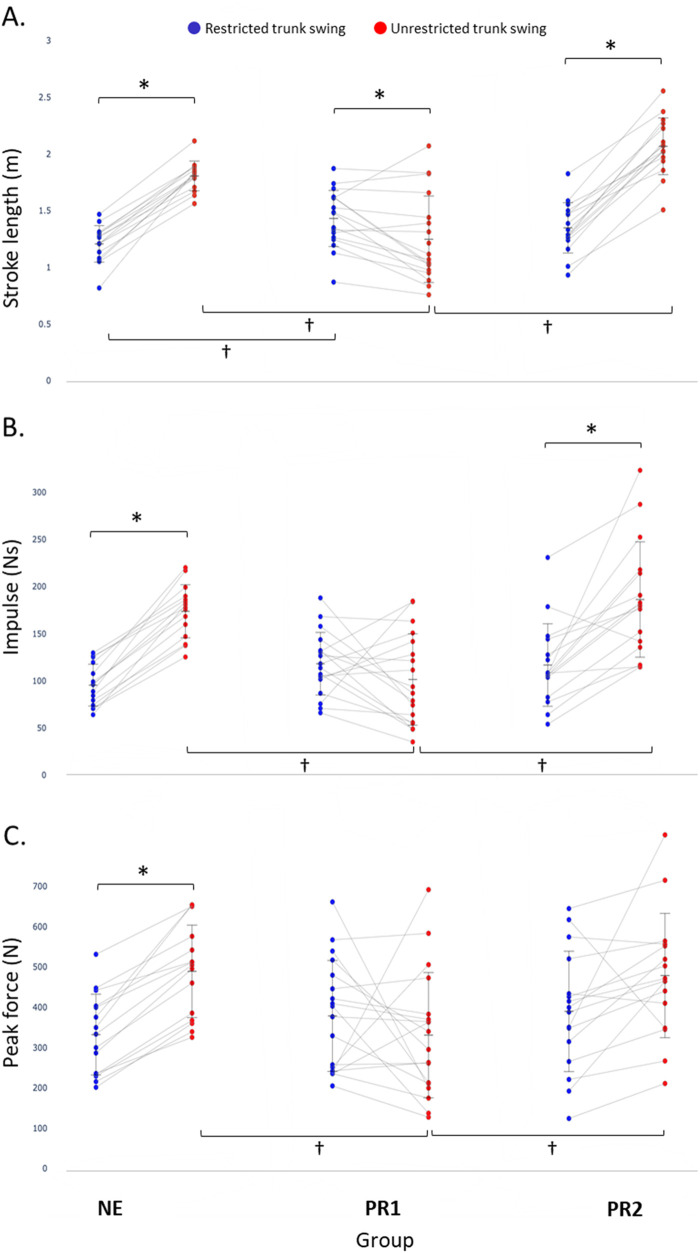
Stroke length **(A)**, peak impulse **(B)**, and peak force **(C)** [black line plots, mean (SD)] during the middle 50% of the 500 m rowing pieces performed in restricted trunk swing (data points, blue circles) and unrestricted trunk swing (data points, red circles) conditions. Between conditions within each group, *P < 0.05. Between-group within each condition, ^†^*P* < 0.05.

There was a significant interaction between condition and group for stroke length, *F*_(2, 46)_ = 81.59, *P* < 0.001, partial *η*^2^ = 0.78. For swing conditions within groups, NE and PR2 rowers demonstrated significantly longer stroke length during the unrestricted trunk swing condition compared with the restricted trunk swing condition (Figure 3A, Table 4). For only the PR1 rowers, stroke length was significantly longer during the restricted trunk swing condition compared with the unrestricted trunk swing condition. Between-group differences were noted in both the unrestricted and restricted trunk swing condition (Figure 3A, red). In the unrestricted trunk swing condition, both NE and PR2 rowers demonstrated significantly longer stroke lengths than PR1 rowers (NE, mean difference ± SE 0.56 ± 0.10 m, *P* < 0.001, 95% CI 0.31–0.91; PR2, 0.82 ± 0.10 m, *P* < 0.001, 95% CI 0.57–1.07). During the restricted trunk swing condition, only the PR1 rowers demonstrated significantly longer stroke lengths than the NE rowers (mean difference ± SE 0.23 ± 0.08 m, *P* = 0.017, 95% CI 0.33–0.42).

**Table 4:** Between unrestricted and restricted trunk swing conditions differences for stroke length (m), stroke impulse (Ns), and peak force (N) in each rower group (PR1, PR2, and NE), mean difference (±SE), and 95% confidence intervals.

Group	Mean difference ± SE	95% CI
Stroke length (meters)		
PR1	−0.18 ± 0.05*	0.08 to 0.29
PR2	0.72 ± 0.06*	0.61 to 0.83
NE	0.60 ± 0.06*	0.49 to 0.71
Stroke impulse (Ns)		
PR1	−16.70 ± 11.37	−39.59 to 6.19
PR2	69.44 ± 12.06*	45.16 to 93.72
NE	78.25 ± 12.46*	53.17 to 103.23
Peak force (N)		
PR1	47.69 ± 40.84	−122.73 to 27.36
PR2	89.06 ± 39.54	9.46 to 168.65
NE	156.92 ± 40.84*	74.12 to 239.13

* *P* ≤ 0.001.

There was a significant interaction between condition and group for stroke impulse, *F*_(2, 46)_ = 20.12, *P* < 0.001, partial *η*^2^ = 0.47. For swing conditions within group, both the NE and PR2 rowers demonstrated significantly greater stroke impulse in the unrestricted trunk swing condition compared with the restricted trunk swing condition (Figure 3B, Table 4). The PR1 rowers showed no significant difference in impulse between conditions (Figure 3B, *P* = 0.149). In the unrestricted trunk swing condition (Figure 3B, red), PR1 rowers demonstrated significantly less stroke impulse than NE (mean difference ± SE −72.25 ± 16.86Ns, *P* < 0.001, 95% CI −114.14 to −30.36) and PR2 (−84.74 ± 16.57Ns, *P* < 0.001, 95% CI −125.91 to −43.57) rowers. There was no significant difference between NE and PR2 rowers in the unrestricted trunk swing condition (mean difference ± SE −12.49 ± 17.33Ns, *P* = 1.00, 95% CI −55.56 to 30.57). There were no significant between-group differences in stroke impulse during the restricted trunk swing condition.

There was a significant interaction between condition and group for peak force (Figure 3C), *F*_(2, 46)_ = 7.26, *P* = 0.002, partial *η*^2^ = 0.24. Within group, NE rowers demonstrated significantly greater peak force in the unrestricted trunk swing condition compared with the restricted trunk swing condition (Figure 3C, Table 4). PR2 rowers demonstrated greater peak force in the unrestricted trunk swing condition compared with the restricted trunk swing condition; however, this did not reach significance (*P* = 0.029). The PR1 rowers showed no significant difference in peak force between conditions. Between-group comparison indicated that, in the unrestricted trunk swing condition (Figure 3C, red), PR1 rowers demonstrated significantly less mean peak force than NE (−158.34 ± 50.29 N, *P* = 0.009, 95% CI −283.29 to −33.40) and PR2 (147.90 ± 49.42 N, *P* = 0.013, 95% CI −270.70 to −25.11) rowers. There was no significant difference between NE and PR2 rowers in the unrestricted trunk swing condition (10.44 ± 51.70 N, *P* = 1.00, 95% CI −118.00 to 138.89). There were no significant between-group differences during the restricted trunk swing condition.

### 3.3 Trunk measurement 2: fixed-seat rowing-specific trunk extension strength measurement

**Figure 4:**
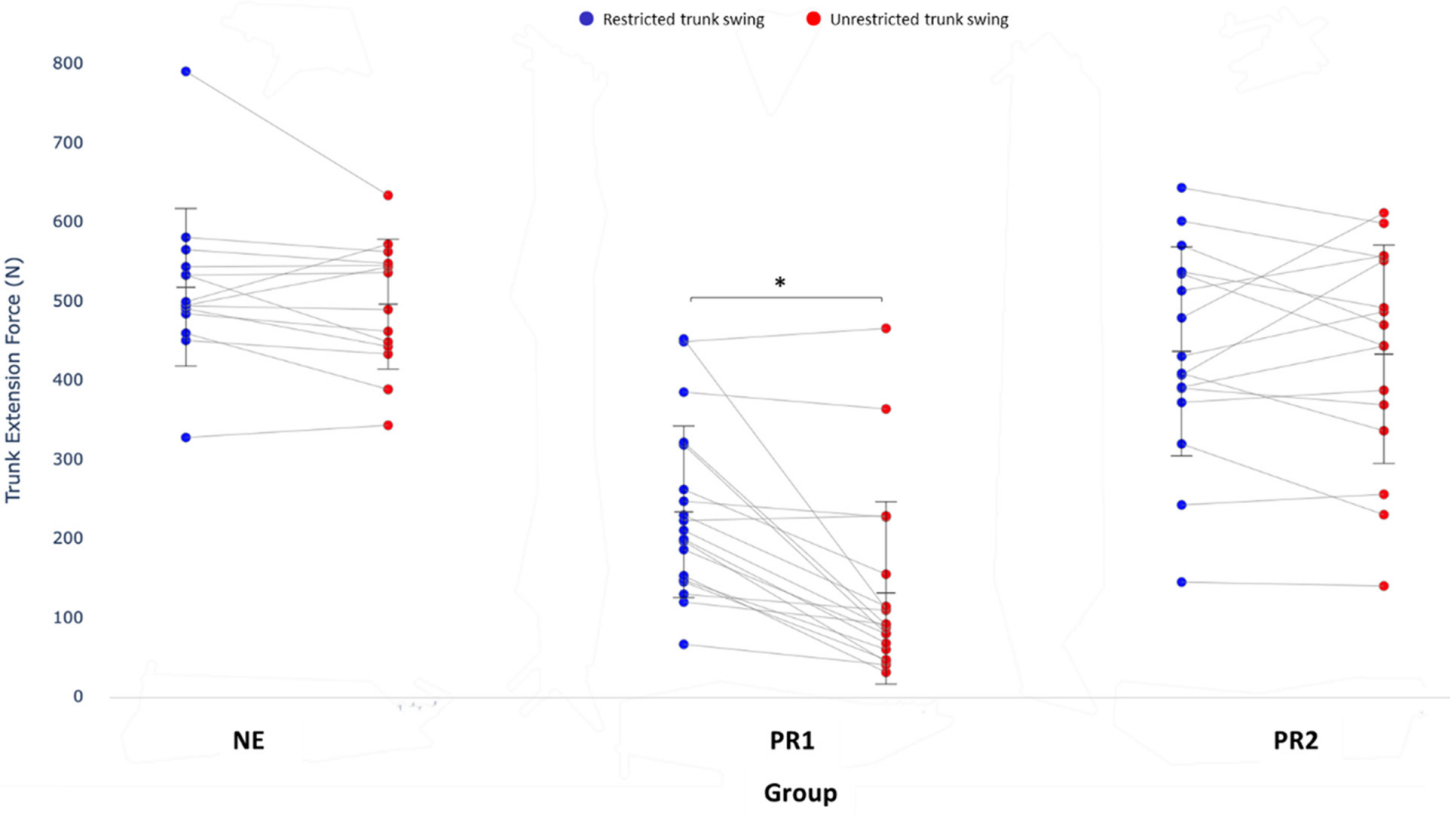
Trunk extension strength [force (N), line plots, mean (SD)] measured in restricted trunk swing (data points, blue circles) and unrestricted trunk swing (data points, red circles) conditions. Between conditions within each group, *P < 0.01.

There was a significant interaction between condition and group for trunk extension force, *F*_(2, 46)_ = 8.01, *P* = 0.001, partial *η*^2^ = 0.26. Only the PR1 rowers generated significantly greater trunk extension force during the supported trunk condition of restricted trunk swing compared with the unrestricted trunk swing condition (Figure 4, mean difference ± SE 102.40 ± 17.92 N, *P* < 0.001, 95% CI 66.32–138.48). There was no significant difference in trunk extension force generated between restricted and unrestricted conditions for NE (21.33 ± 20.88 N, *P* = 0.312, 95% CI −20.70 to 63.36) and PR2 (3.58 ± 19.53 N, *P* = 0.856, 95% CI −35.74 to 42.89) rower groups.

## 4 Discussion

There is a difference in the impact of trunk swing restriction on performance measures and trunk extension strength among PR1 rowers compared with both PR2 and NE rowers. While the restricted trunk swing condition resulted in a faster time to complete 500 m, minimal impact on force production, and improved trunk extension strength for PR1 rowers, the restricted trunk swing condition decreased rowing performance for PR2 and NE rowers and did not improve trunk extension strength in sitting. These findings demonstrate the differentiation between PR1 and PR2 rower groups with respect to the contribution of trunk swing during fixed-seat rowing and provide evidence-based reasoning for the classification of fixed-seat rowers.

The increased stroke length shown by PR2 and NE rowers in the unrestricted trunk swing condition compared with the restricted trunk swing condition suggests that this condition facilitated the rowers to incorporate increased excursion of trunk swing, resulting in the increased length of the stroke. Conversely, the PR1 group showed an increase in stroke length in the restricted trunk swing condition. The trunk strap provided trunk support that allowed PR1 rowers a greater degree of forward flexion through the spine by leaning over the top of the strap that facilitated increased stroke length and better trunk support needed for a stronger arm pull during the drive. As stroke length contributes to the force generated during the drive (16), the inability to increase the use of trunk swing about the hip limits these performance variables in PR1 rowers during the unrestricted trunk condition.

Comparison between para rowers specifically (PR1 and PR2 rower groups) revealed significant differences in the strength of the hip that is likely to influence the between-group differences in performance of trunk swing in rowing. In previous research, kinematic measurements of unimpaired rowers during the drive portion of the rowing stroke demonstrated that very little movement occurs through the spinal segments, and the main segment of motion of the trunk is the pelvis, indicating the hip joint as the fulcrum for trunk swing (17). The motor pattern associated with trunk swing in unimpaired rowers is described as the spinal extensor muscles (e.g., erector spinae muscles and latissimus dorsi) working to maintain a somewhat rigid lever of the trunk while the hip extensors (e.g., biceps femoris and gluteus maximus) extend the trunk by extension (posterior rotation) of the pelvis (17). While no research has examined electromyography in para rowers, based on the hip function strength demonstrated for the PR2 group in the current study and the lack of differences between PR2 and NE group across all performance metrics, it can be hypothesized that PR2 rowers performed trunk extension in a similar manner to unimpaired rowers (NE).

PR1 rowers are not without any trunk movement. However, it is likely that extension through the spine noted in PR1 rowers is driven by muscle groups about the spine (i.e., latissimus dorsi and erector spinae muscles), not by hip extensors. The muscles of the spine typically serve as stabilizers during the rowing stroke (17) and show less contribution toward force production in trunk swing compared with the hip extensor muscles.

These results agree with recommendations that when classifying seated athletes, factors such as trunk and hip range of motion/strength and impact of seating configuration on trunk strength should be the principal determinants of class (2). Furthermore, these results suggest that PR classification may consider the inclusion of hip strength thresholds explicitly as factors of classification of fixed-seat rowers in conjunction with the current use of trunk swing test and observation on the rowing ergometer. Potentially establishing a threshold based on hip strength [e.g., at the level of muscle activation against gravity (3/5)] may serve to further differentiate PR1 and PR2 fixed-seat sport classes in PR and provide an additional metric informing classification of fixed-seat rowers.

Larger impulse is associated with more power generation over the duration of the rowing stroke, thereby allowing improved rowing performance (18). Despite the significant (albeit slight) increase in stroke length in the supported condition, PR1 rowers did not show a significant increase in stroke impulse in this condition. Conversely, PR2s and NE rowers displayed increased stroke length and impulse when trunk swing was unrestricted. Adding trunk swing allowed these rowers to produce force over a larger range of motion and likely recruit their hip extensor muscles for trunk swing. This allowed for force application over a longer period of time (greater impulse) and improved rowing performance. Seated athletes lacking motor control of the pelvis often adopt a posture that features a posterior pelvic tilt/retroversion (19). This pelvic retroversion allows for a stable base upon which an athlete's upper extremity can move with force and athleticism but limits trunk range of motion (19). In the present study, this may explain how the PR1 rowers showed similar levels of performance when the trunk was unsupported during the unrestricted trunk swing condition compared with the restricted trunk swing. It is likely that PR1 rowers adopt a similar posture during the unrestricted trunk swing condition which serves to stabilize to prevent falling forward when reaching toward the catch and may further provide an explanation for the decrease in stroke length and therefore impulse generation.

Importantly, the primary reason a seated athlete would adopt the use of strapping is due to impaired trunk function to maintain stability (2). Specifically, wheelchair athletes with increased neurological deficits about the trunk require greater trunk stabilization to maintain stable sitting posture while producing force with their upper extremities and performing sport-specific tasks (20). In the current study, the trunk strap appeared to have facilitated increased trunk extension force production by ensuring stability in PR1 rowers and therefore the ability to maximize the use of trunk extensor muscles without risk of falling forward in the seated position. This contrasts with the PR2 and NE rowers that did not show any impact of trunk strapping on trunk extension force produced. NE and PR2 rowers have means of internal stability (through muscle function about the lower trunk and hips) to aid production of trunk extension force pivoting about the hip, as is performed in rowing, without risk of loss of sitting balance.

When the trunks of all investigated rowers were stabilized, there was no difference in rowing performance among the three groups (the mean time over 500 m was 157 s). Despite differences in trunk function and motor impairment, PR1 rowers managed to be competitive with PR2 and NE rowers with an even lower rate of strokes per minute. While rib stress fractures are sport-specific injuries of a high-performance rowers (21), the high external forces of the trunk strap applied to the rib cage of the PR1 rowers (when trunk strap fixation was mandatory for PR1 rowers) during training and racing have been suggested to contribute to risk of rib stress fractures among PR1 rowers (22). Importantly, imposing restrictions on sport-specific function beyond that incurred as a result of a physical disability does not align with the Paralympic movement (7, 8).

### 4.1 Limitations

A limitation of this study is that while all PR1 and PR2 rowers competing at WR Gavirate International Para Rowing Regattas, May 2018 and May 2019, were invited to participate, some rowers were inevitably missed, as participation was voluntary. Additionally, testing was performed on a dryland rowing ergometer and not on water in a rowing shell. Rowing shells have reduced lateral stability compared with ergometers, posing a greater challenge to sitting balance. However, this study followed the WR Classification protocols, which evaluate rowers on stationary ergometers rather than in a rowing shell on the water. This allows for more standardized evaluation during classification; therefore, ergometers were appropriate for the purpose of this study. Performance variables were collected over 500 m; however, para rowers typically race over 2,000 m. Due to testing of the participants during an international regatta, 2 × 500 m pieces were chosen to simulate different aspects of a race piece and not impact their performance at the regatta. Finally, while rowing experience and training data are reported for each group, the potential impact of these variables on performance was not controlled statistically.

A final limitation in this study is that we used an internal calculation of stroke length from the Concept2 PM4 monitor directly. The PM4 uses the sprocket radius and the number of flywheel rotations for this calculation. It is important to note that there can be as much as one-third revolution uncertainty in this calculation. However, all PR1 and PR2 rowers were tested on the same ergometer for both conditions, and all NE rowers were tested on the same ergometer for both conditions.

### 4.2 Conclusion

The ability, or lack thereof, to incorporate functional trunk swing into the rowing stroke underpins the theoretical construct that functionally defines PR1 and PR2 rowers during classification in PR. The results of the present study provide empirical performance data supporting the differentiation in the contribution of trunk swing to rowing performance between PR1 and PR2 rowers. Future research should continue to investigate the impact of seating, impairments of trunk strength, range of motion and coordination on peak force, impulse generation, and other core determinants of fixed-seat rowing performance to further inform PR classification.

## Data Availability

The raw data supporting the conclusions of this article will be made available by the corresponding author(s), without undue reservation.
